# The “can do, do do” concept in individuals with chronic obstructive pulmonary disease: an exploration of psychological mechanisms

**DOI:** 10.1186/s12931-021-01854-1

**Published:** 2021-10-06

**Authors:** J. Carl, K. Schultz, T. Janssens, A. von Leupoldt, K. Pfeifer, W. Geidl

**Affiliations:** 1 Department of Sport Science and Sport, Friedrich-Alexander University Erlangen-Nürnberg, Gebbertstraße 123b, 91058 Erlangen, Germany; 2 Klinik Bad Reichenhall, Centre for Rehabilitation, Pneumology, Orthopaedics, Salzburger Str. 8 – 11, 83435 Bad Reichenhall, Germany; 3 Research Group on Health Psychology, Katholieke Universiteit Leuven, Tiensestraat 102, Box 3726, 3000 Leuven, Belgium

**Keywords:** Chronic obstructive pulmonary disease, Physical activity, Capacity, Exercise

## Abstract

**Background:**

The “can do, do do” concept aims at identifying subgroups among persons with chronic obstructive pulmonary disease (COPD). Following a two-dimensional categorization, individuals are binarily classified with respect to their levels of physical capacity (“can’t do” or “can do”) and physical activity (“don’t do” or “do do”), resulting in four disjunct quadrants. The approach has been debated recently and the latest articles have concluded that the quadrants should be specifically examined in terms of psychological aspects of physical activity. Therefore, the goal of the present study was to explore the role of psychological variables in physical activity in the context of the “can do, do do” quadrant concept.

**Methods:**

Within the scope of secondary data analyses of the “Stay Active After Rehabilitation” (STAR) randomized controlled trial, a total of 298 COPD rehabilitants of an inpatient pulmonary rehabilitation program were grouped into the suggested quadrants. We set fixed cut-offs at 70% of relative 6-min walking test performances for healthy individuals (physical capacity dimension) and 5.000 steps per day (physical activity dimension). Univariate and multivariate logistic regression analyses served to analyze whether depression scores, fear avoidance behaviors, disease-specific anxiety, self-concordance for physical activity, and five indicators of physical activity-related health competence (PAHCO) effectively discriminated between the “don’t do” and “do do” groups.

**Results:**

Among persons with lower relative physical capacity, depression scores, fear avoidance behaviors, and disease-specific anxiety (univariate case) significantly differentiated between the more and the less active. Among persons with higher relative physical capacity, fear avoidance behaviors, disease-specific anxiety, as well as three PAHCO indicators (physical activity-specific self-efficacy, self-control, and affect regulation) significantly separated the more and the less active. In multivariate analyses, only fear avoidance behaviors and affect regulation discriminated among individuals with better relative physical capacity.

**Conclusion:**

The findings identified important psychological and competence-oriented variables that explain discrepancies in the quadrant concept. Based on this, we discuss implications for physical activity promotion in individuals with COPD. Respiratory research can benefit from future studies complementing the quadrant concept through further behavioral analyses.

*Trial registration* Clinicaltrials.gov, ID: NCT02966561. Registered 17 November, 2016, https://clinicaltrials.gov/ct2/show/NCT02966561.

## Introduction

Chronic obstructive pulmonary disease (COPD) ranks in first place among the most prevalent respiratory indications and causes one of the highest mortality rates worldwide [1, 2]. Studies have consistently shown that physical activity indicators have the strongest predictive power regarding all-cause mortality in persons with COPD [3, 4]. In this context, physical activity interventions that follow an individually centered and individually tailored approach are recommended [5, 6]. To facilitate the specification of adequate intervention content, it has been considered valuable to identify subgroups of physical activity among individuals with COPD [6–8].

In this respect, Koolen et al. [7] suggested categorizing individuals with COPD along two dimensions. One dimension characterizes the *relative physical capacity* (PC) of individuals and reflects their ability to perform physical activities. Based on a cut-off value, the researchers applied a dichotomy, describing individuals who have the ability to perform physical activities (“can do”) on the one hand, and individuals who do not have the ability (“can’t do”) on the other. The second dimension represents the actual *physical activity* (PA) of persons with COPD. Depending on the achievement of an average of 5.000 steps per day, the authors classified individuals as performing sufficient habitual PA (“do do”), on the one hand, or insufficient habitual PA (“don’t do”), on the other. Although it is not explicitly stated, the researchers followed the suggestion of the International Classification of Functioning, Disability and Health (ICF) framework [9, 10] to consider both (physical) capacity and performance when targeting people’s functioning [11]. When plotting individuals along these two axes, the classification results in a quadrant-like visualization (see Fig. 2). According to the concept, particular attention should be paid to individuals who do not show a convergence in both indicators. From a practical perspective, this classification has the potential to highlight “discrepancies” [7] in individuals who actually “can do” but “don’t do” (Quadrant 2) or “can’t do” but “do do” (Quadrant 3).

These discrepancies raise the question of why several individuals seem to underperform (Quadrant 2) or overperform (Quadrant 3) relative to their PC. Explaining these discrepancies in the quadrant concept may enhance our understanding of physical functioning and support adequate referral to exercise-based care. Previous attempts primarily used clinical characteristics to explain these discrepancies. Koolen et al. [7] stated that the PC–PA based quadrants differ considerably in multiple clinical characteristics, such as forced expiratory volume in one second (FEV_1_), the COPD Assessment Test (CAT), and GOLD indicators. On the contrary, on the basis of longitudinal analyses, Sievi et al. [12] concluded that “there are no clinical characteristics allowing to distinguish between the PC–PA quadrants” (p. 1) and that scientific evidence regarding long-term health outcomes is scarce. Nevertheless, both groups of researchers congruently highlighted the urgency of shedding more light on the quadrants of the “can do, do do” concept. More specifically, Van’t Hul et al. [13] assumed that “it is likely that the main determinant(s) of the low habitual physical activity needs to be found in the behavioral aspects” (p. 1). Similarly, Sievi, Kohler, and Clarenbach [14] concluded in a response that “to disentangle behavioral and physical aspects of inactivity will be the challenge of future research” (p. 2).

In this regard, insights from the scientific disciplines of rehabilitation and exercise psychology offer a plethora of potential explanations for the two discrepancy phenomena. For instance, individuals could suffer from symptoms of depression or disease-specific anxiety [15, 16], which may deter them from being physically active [17, 18]. Persons with COPD may cultivate catastrophizing cognitions and fear avoidance beliefs, resulting in avoidance of PA [19]. Moreover, positive attitudes toward PA and motivational mechanisms (e.g., the adoption of autonomous forms of motivation) may play a central role in initiating and maintaining PA behaviors [20]. From a competence-oriented perspective, the lack of ability to adequately align activities with respect to physical health (control of physical load) and psychological well-being (affect regulation) may prevent individuals with COPD from leading a physically active lifestyle [21, 22]. Finally, the causes of discrepancy may also lie in self-regulatory (volitional) skills that are required to turn PA intentions into action [23]. Due to the breadth of research and the number of approaches in the area of behavior change, it is hardly possible to outline all plausible mechanisms and constructs of the PA behavior (for an overview, see [24–26]). However, both research and practice can benefit from exploring those psychological variables which promote an understanding of the PC–PA quadrant concept.

### Aims and research questions

Using secondary data analyses, the goal of the present study was to (a) replicate the PC–PA quadrant concept in a sample of individuals with COPD undergoing inpatient rehabilitation and (b) explore the role of psychological variables in discriminating between individuals who “don’t do” and “do do.” In the present study, we chose an exploratory approach because the selection of adequate variables was made after the initial publication of the PC–PA quadrant concept [7] and, hence, after the definition of the main analyses of the “Stay Active After Rehabilitation” (STAR) study [27]. Accordingly, we formulated no specific hypotheses for the present study.

## Methods

### Study design and participants

The STAR study (Clinical Trials Registration Number: Clinicaltrials.gov, ID NCT02966561) is located within the setting of inpatient pulmonary rehabilitation and adds a pedometer-based behavioral intervention to standard care by employing a randomized controlled research design [27]. The study consisted of five measurement time points: 2 weeks before inpatient rehabilitation (T0), right at the beginning (T1) and the end (T2) of inpatient rehabilitation, as well as 6 weeks (T3) and 6 months (T4) after the rehabilitation stay. The application of accelerometry and a six-minute walking test at baseline (T0/T1) enabled our research team to reproduce the quadrant concept with the same operationalization of PC as that undertaken by Koolen et al. [7]. Importantly, the STAR study encompasses a combination of physical, psychological, and behavioral assessments, allowing to complement the quadrant concept with further behavioral aspects of PA. In line with the criteria defined in the main analyses of STAR [27, 28], the present study included all participants who (a) provided informed consent to participate in the study, (b) were granted inpatient rehabilitation at Bad Reichenhall Clinic, Germany, (c) actually attended pulmonary rehabilitation (PR), and (d) whose COPD diagnosis was confirmed by a lung function test (Tiffeneau index FEV1/VC ≤ 0.70) that was performed at the beginning of the PR (T1). In line with the analytical strategy of the present article, participants (e) had to provide valid accelerometry-measured PA data at T0 and valid PC data at T1. Post-rehabilitation data of STAR (T2, T3, T4) was not used for this study.

### Material

To determine *relative PC*, we applied the same procedure as that undertaken by Koolen et al. [7]. First, we registered individuals’ distances of a six-minute walking test (6MWT) which was conducted at the beginning of the rehabilitation stay (T1). Afterwards, these performances were set in relation to age-, sex-, height-, and weight-adjusted reference values (pred_capacity) by relying on a formula for healthy subjects published by Troosters, Gosselink, and Decramer [29]. This quotient (6MWT/pred_capacity) served to classify individuals based on a cut-off value of 70% (“can do”: ≥ 70%; “can’t do”: < 70%) [7].

*PA levels* were measured objectively with the validated ActiGraph (Pensacola, Florida) wGT3X-BT accelerometer [30]. Two weeks prior to rehabilitation (T0), each study participant was asked to wear the device for at least ten hours per day for seven consecutive days. The technical settings for accelerometry followed COPD-specific recommendations [31] and can be retrieved in detail from the study protocol [27] or a baseline analysis of physical activity and sedentary behavior patterns [8]. The average number of steps per day served as the primary outcome with a fixed cut-off value of 5.000 steps per day [7, 32].

All psychological indicators (*n* = 10) were assessed via paper- and pencil questionnaires in the German language, providing validated sum scores (T1). The PHQ-D Questionnaire with its 10 items (α = 0.86) was employed to measure individuals’ *depressive symptoms* [33]. The COPD-Anxiety-Questionnaire (CAF) [34] assessed patients’ *disease-specific anxiety* with 27 items. Although it contains five subscales (0.80 ≤ α ≤ 0.92), the instrument enables the aggregation of a sum score. Moreover, we used the Fear-Avoidance Beliefs Questionnaire for COPD (FAB) which encompassed a total of 12 items measuring *fear* and *avoidance of physical activities* (α = 0.93) [35]. The Breathlessness Catastrophizing Scale (BCS) was used to measure *negative cognitions toward breathlessness* and the perceived inability to control them [36]. The instrument contained 13 items and showed a Cronbach’s α of 0.94 in our study. *Self-concordance* expresses the degree to which a PA-related goal represents personal interests and values. In line with self-determination theory, the Self-Concordance Scale (SSK) [37] included four subscales, each with three items (0.72 ≤ α ≤ 0.90). The scales intrinsic motivation and identified motivation contributed positively, while introjected and extrinsic motivation contributed negatively to the sum score. The remaining five constructs had a competence-oriented character and were taken from the Physical Activity-related Health Competence (PAHCO) Questionnaire [22, 23], which has been extended and specifically validated for persons with COPD recently [23]: *PA-specific self-efficacy* (three items, α = 0.83), *control competence for physical load* (six items, α = 0.84), *control competence for affect regulation* (four items, α = 0.88), *PA-specific self-control* (three items, α = 0.84), and *emotional attitudes towards PA* (four items, α = 0.89). We drew on *disease-related indicators* to describe the whole sample and its subgroups, including the COPD Assessment Test (CAT) [38], GOLD classification [A/B/C/D], the Saint-George’s Respiratory Questionnaire (SGRQ) [39], and the Tiffeneau index (FEV_1_/VC) reflecting people’s lung function (after bronchospasmolysis). The FEV1%_pred index, in turn, served to derive the GOLD stages (I–IV) indicating disease severity. Finally, we acquired additional *person-related information*, such as age, gender, body mass index [kg/m^2^], employment, comorbidities, and smoking status.

### Data analysis

We initially assigned the included participants to the four quadrants. The comparison between the four subgroups was grounded on Multivariate Analysis of Variance (MANOVA) for continuous variables (including Bonferroni post-hoc tests) or *χ*^2^ tests for categorical variables. We computed univariate logistic regression models with each of the ten psychological indicators to contrast the individuals who were categorized as “don’t do” or “do do” at baseline. In this context, we conducted separate analyses of the two groups with a lower relative PC (“can’t do”) and a higher PC (“can do”). Subsequently, we calculated multivariate logistic regression models in which all ten psychological indicators were included simultaneously to get an indication of the most relevant variables. Nagelkerke’s R^2^ statistics were retrieved to determine the explanatory power of the entire multivariate model. We treated missing data by applying expectation maximization (EM) based imputation techniques (5.4% of all cases). The data management was performed in SPSS (Version 25, IBM, Armonk, USA), while we ran all statistical analyses with R (Version 4.0.4, R Core Team). Due to the exploratory nature of this study, we set the significance level at *p* < 0.05.

## Results

### Study participants and the PC–PA quadrants

**Fig. 1:**
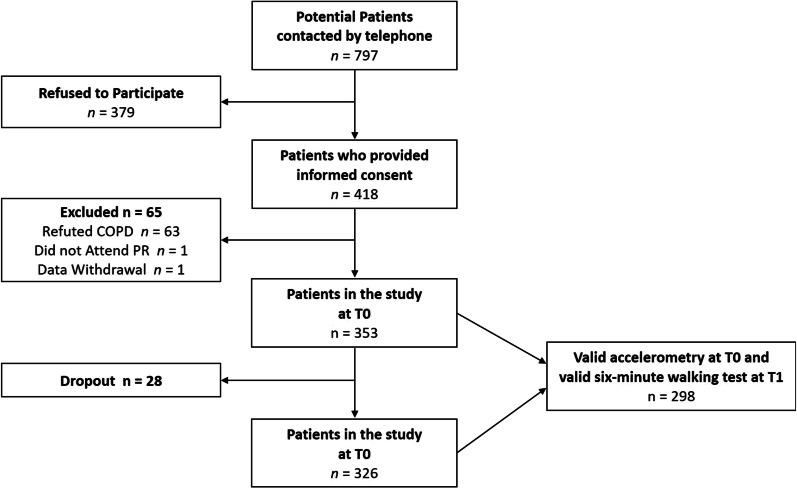
Study flow

**Fig. 2:**
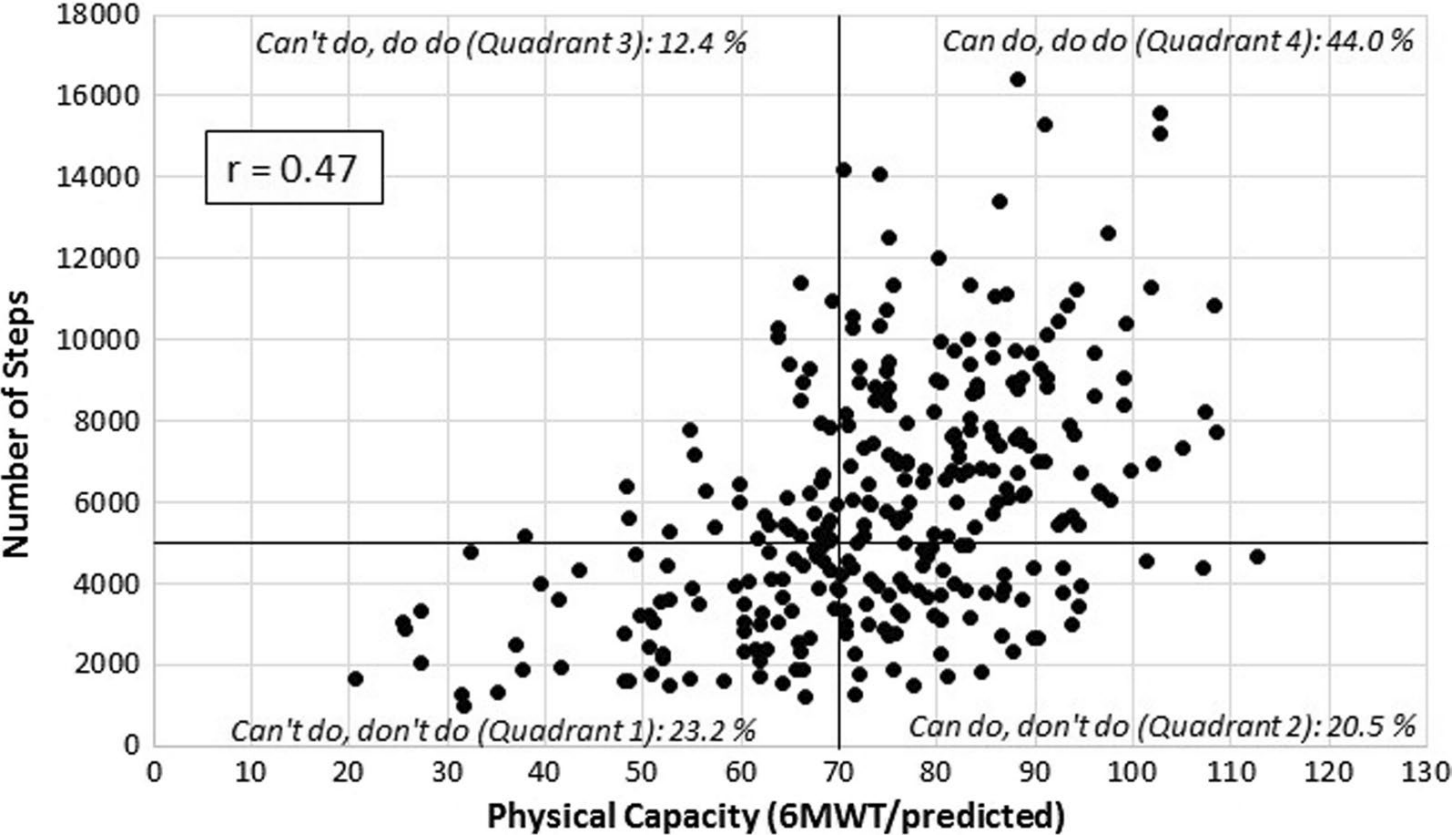
The physical capacity–physical activity quadrant concept with the baseline data of the STAR study. Abbreviations: 6MWT = Six-Minute Walking Test, STAR = Stay Active After Rehabilitation [study]

The participant flow for the present study can be found in Fig. 1. A total of *N* = 298 participants were finally included in the analyses. Linear regression revealed a significant association between relative PC and number of steps: *β* = 0.47 [95% CI: 0.37–0.57], *p* < 0.001. Following the quadrant concept (Fig. 2), *n* = 69 (23.2%) individuals with COPD were classified as “can’t do, don’t do” (Quadrant 1), *n* = 61 (20.5%) as “can do, don’t do” (Quadrant 2), *n* = 37 (12.4%) as “can’t do, do do” (Quadrant 3), and *n* = 131 (44.0%) as “can do, do do” (Quadrant 4). A description of the whole sample and the four quadrants can be retrieved from Table 1. The four groups differed in terms of lung function, age, gender, disease severity, body mass index, disease-related consequences, employment status, and perceived quality of life.

**Table 1:** Characterization of the sample and the quadrants

	Whole Sample	Quadrant 1 (“can’t do, don’t do”)	Quadrant 2 (“can do, don’t do”)	Quadrant 3 (“can’t do, do do”)	Quadrant 4 (“can do, do do”)	Comparison between subgroups	Significant compa-risons or direction
N	298	69	61	37	131	–	–
Age [in years]	58.13 ± 5.48	60.01 ± 7.00	58.87 ± 5.38	56.68 ± 5.55	57.21 ± 4.18	*F* (3, 294) = 5.41, *p* = 0.001, *η*^2^ = 0.05	Q1, Q2 > Q3, Q4
Gender Distribution	30.5% female, 69.5% male	43.5% female, 56.5% male	23.0% female, 77.0% male	37.8% female, 62.2% male	25.2% female, 74.8% male	*χ*^2^ (3) = 9.80, *p* = 0.02	Men were more strongly represented in Q2 and Q4
Body mass index (BMI) [kg/m^2^]	27.44 ± 6.57	26.66 ± 7.17	29.43 ± 6.97	26.52 ± 6.82	27.19 ± 5.81	*F* (3, 294) = 2.53, *p* = 0.057, *η*^2^ = 0.03	none
GOLD Stages [1/2/3/4]	27/130/114/24	1/14/37/16	4/28/27/2	1/16/14/5	21/27/36/1	*χ*^2^ (9) = 66.3, *p* < 0.001	Higher stages in Q1, lower stages in Q4
GOLD Classification [A/B/C/D]^a^	4/126/0/148	0/26/0/40	1/25/0/29	0/26/0/40	3/63/0/57	*χ*^2^ (3) = 4.77, *p* = 0.19	None
Lung Function (FEV_1_ pred)	53.48 ± 18.25	39.62 ± 13.25	53.37 ± 16.21	49.58 ± 17.59	61.87 ± 16.91	*F* (3, 291) = 29.3, *p* < 0.001, *η*^2^ = 0.23	Q1 < Q2, Q3 < Q4
COPD Assessment Test (CAT)	23.29 ± 6.67	26.36 ± 6.41	23.85 ± 6.47	24.58 ± 6.04	21.05 ± 6.34	*F* (3, 284) = 11.1, *p* < 0.001, *η*^2^ = 0.11	Q1, Q2, Q3 > Q4
Quality of Life (SGRQ)	52.66 ± 10.40	46.35 ± 9.94	51.97 ± 9.84	52.85 ± 10.63	56.36 ± 9.17	*F* (3, 277) = 15.1, *p* < 0.001, *η*^2^ = 0.14	Q1 < Q2, Q3 < Q4
Smoking Status	46.2% current smokers, 53.8% no/ former smokers	46.8% current smokers, 53.2% no/ former smokers	57.6% current smokers, 42.4% no/ former smokers	48.6% current smokers, 51.4% no/ former smokers	39.7% current smokers, 60.3% no/ former smokers	*χ*^2^ (3) = 5.26, *p* = 0.15	None
Employment Status	60.8% full-time, 15.8% half-time, 9.5% pension, 15.8% not employed	49.2% full-time, 13.1% half-time, 23.0% pension, 13.1% not employed	47.3% full-time, 10.9% half-time, 12.7% pension, 10.9% not employed	51.5% full-time, 24.2% half-time, 3.0% pension, 24.2% not employed	75.0% full-time, 16.9% half-time, 3.2% pension, 16.9% not employed	*χ*^2^ (9) = 46.8, *p* < 0.001	Unemployment was overrepresented in Q2, full-time employment in Q1, and pension in Q1
Number of Comorbidities	4.66 ± 2.50	4.17 ± 2.77	4.67 ± 2.33	4.81 ± 2.64	4.88 ± 2.38	*F* (3, 294) = 1.25, *p* = 0.293	None
Average Number of Steps per Day	5850 ± 3043	2991 ± 1104	3510 ± 945	6799 ± 1839	8323 ± 2370	*F* (3, 294) = 171.7, *p* < 0.001, *η*^2^ = .64	Q1 < Q3 < Q4, Q2 < Q3 < Q4
6-minute Walking Test [m]	449.8 ± 102.3	326.8 ± 83.0	481.1 ± 59.9	392.5 ± 59.5	516.3 ± 61.5	*F* (3, 294) = 135.6, *p* < 0.001, *η*^2^ = 0.58	Q1 < Q3 < Q2 < Q4

^a^For the inference statistical group comparison, we removed the cases of the GOLD Classifications A and C due to the low number of individuals in these categories (for the *χ*^2^ test)

Q1, quadrant 1; Q2, quadrant 2; Q3, quadrant 3; Q4, quadrant 4

### Univariate analyses

Among the participants who “can’t do” (*n* = 106; 35.6%), three psychological variables significantly distinguished between COPD rehabilitants who “don’t do” (Quadrant 1) and those who “do do” (Quadrant 3). Individuals achieving more than 5.000 steps per day showed fewer depressive symptoms, lower disease-specific anxiety, and fewer fear avoidance behaviors. The remaining seven variables did not differentiate between these two groups (Table 2). In contrast, five variables significantly discriminated between the individuals who “don’t do” and “do do” among those who “can do” (*n* = 192; 64.5%). Individuals with COPD who did more than 5.000 steps per day demonstrated less disease-specific anxiety and fear avoidance behaviors compared to those who did not (< 5.000 steps/day). In addition, they reported better PA-specific self-efficacy, self-control, and control competence for affect regulation.

**Table 2:** Univariate comparison of the quadrants in terms of psychological and behavioral aspects of physical activity using logistic regression models

Variable	Quadrant 1 versus Quadrant 3				Quadrant 2 versus Quadrant 4			
	*b*	*Wald*	*p*	*exp*	*b*	*Wald*	*p*	*exp*
Depression	−0.10	4.07	0.044*	0.91	−0.04	1.41	0.235	0.96
COPD Anxiety	−0.03	4.85	0.028*	0.97	−0.02	4.23	0.040*	0.98
Fear Avoidance Behavior	−0.05	5.88	0.015*	0.95	−0.06	11.8	< 0.001**	0.95
Breathlessness Catastrophizing	−0.02	1.27	0.260	0.98	−0.02	2.19	0.139	0.98
Motivation/Self-Concordance	0.03	1.26	0.262	1.03	0.03	1.37	0.242	1.03
PA-specific Self-Efficacy	0.10	0.228	0.632	1.11	0.37	4.13	0.042*	1.44
Control of Physical Load	0.16	0.426	0.514	1.17	0.33	3.08	0.079	1.39
PA-specific Affect Regulation	0.14	0.449	0.503	1.15	0.60	10.9	< 0.001*	1.82
PA-specific Self-Control	0.10	0.247	0.619	1.10	0.32	4.46	0.035*	1.38
Emotional Attitude towards PA	0.10	0.736	0.391	1.11	0.19	3.31	0.069	1.20

*p < 0.05, **p < 0.01

### Multivariate analyses

When considering all ten psychological variables simultaneously (Table 3), the analyses contrasting the two “can’t do” groups revealed that no variable took a dominant role in predicting less or more active individuals (Nagelkerke’s R^2^ = 0.124). However, the multivariate regression model for the “can do” quadrants indicated that fear avoidance behaviors (*b* = −0.06, *Wald* = 6.09, *p* = 0.014) and control competence for affect regulation (*b* = 0.50, *Wald* = 4.27, *p* = 0.039) remained significant among the variables (Nagelkerke’s R^2^ = 0.153).

**Table 3:** Multivariate comparison of the quadrants in terms of psychological and behavioral aspects of physical activity using multiple logistic regression

Variable	Quadrant 1 versus Quadrant 3				Quadrant 2 versus Quadrant 4			
	*b*	*Wald*	*p*	*exp*	*b*	*Wald*	*p*	*exp*
Depression	−0.05	0.613	0.434	0.95	−0.01	0.040	0.842	0.99
COPD Anxiety	−0.02	0.838	0.360	0.98	0.00	0.064	0.800	1.00
Fear Avoidance Behavior	−0.05	1.68	0.101	0.95	−0.06	6.09	0.014*	0.94
Breathlessness Catastrophizing	0.03	1.26	0.262	1.03	0.01	0.092	0.761	1.01
Motivation/Self-Concordance	0.02	0.165	0.684	1.02	−0.02	0.644	0.422	0.98
PA-specific Self-Efficacy	0.01	0.001	0.974	1.01	0.11	0.285	0.593	1.12
Control of Physical Load	0.12	0.094	0.759	1.13	−0.13	0.261	0.609	0.88
PA-specific Affect Regulation	−0.01	0.000	0.985	0.99	0.50	4.27	0.039*	1.64
PA-specific Self-Control	−0.17	0.267	0.605	0.84	0.27	1.69	0.194	1.31
Emotional Attitude towards PA	−0.06	0.112	0.738	0.94	−0.11	0.599	0.439	0.89

*p < 0.05

## Discussion

Conceptualized as an exploratory study based on secondary data analysis from the STAR study, this article followed recent calls [13, 14] pleading for an examination of behavioral variables in the context of the PC–PA quadrant concept for persons with COPD [7]. The present study revealed that, in individuals displaying lower relative PC levels, lower depression scores, lower disease-specific anxiety, and lower fear avoidance behaviors univariately discriminated between more and less physically active rehabilitants with COPD. Similarly, the PA behavior of individuals with higher relative PC levels was significantly predicted by disease-specific anxiety and fear avoidance behaviors. In this group, conversely, also three competence-oriented indicators (PA-specific self-efficacy, self-control, and affect regulation) effectively distinguished between the more and the less active. However, when applying multivariate analyses, only fear avoidance behaviors and PA-specific affect regulation for individuals with a higher relative PC remained significant as discriminatory variables.

Aiming for an individually tailored approach to PA promotion, the “can do, do do” concept was originally introduced to identify relevant subgroups among persons with COPD. Practical efforts may prioritize intervention content differently in accordance with individuals’ PC levels. Indeed, rehabilitation in the Netherlands has already integrated the quadrant concept in the referral model for patients with COPD, with the resulting profiles guiding exercise-based care [6]. Our findings indicate that, in persons with a lower relative PC, it may be promising to support the improvement of depressive symptoms, fear avoidance behaviors, and disease-specific anxiety. In this respect, reviews suggest that practitioners could draw on cognitive restructuring, behavioral confrontation, or education elements as useful intervention content [40–42]. This does not mean that PA and exercise promotion should only start *after* the successful treatment of these negative conditions and barriers to PA. Rather, systematic reviews consistently underscore that exercise interventions can make a substantial contribution to the reduction of depression and anxiety in persons with COPD [41, 43, 44], especially when these emphasize the integrated nature of body and mind or are provided together with psychological content [45, 46]. Taken together, the findings of the present study suggest that PA and exercise interventions should be considered an essential part of COPD treatment, even in less physically capable individuals. For this group, it appears crucial to design exercise interventions right from the beginning in a way that psychological variables are purposefully integrated and influenced simultaneously. In persons with better relative PC, we registered similar effects of fear avoidance and disease-specific anxiety. However, the significant effects of competence variables imply that alongside targeting fear avoidance and disease-specific anxiety, a stronger focus on positive constructs (i.e., individual resources) may be effective for the “can do” subgroups. In this regard, the practical recommendations of the PAHCO model suggest not only focusing on functional exercise, but also interlocking training with motor learning, cognitive activation, and positive affective experiences to achieve beneficial health outcomes in the long run [21].

From the perspective of the quadrant concept, the present study could basically reproduce the association between relative PC and PA, with the *β* value of 0.40 found in earlier studies [7] lying inside the confidence interval of the present study. Despite applying the same operationalization to calculate relative PC, our study included more participants (60.5%) in the “can do” categories (Quadrants 2 and 4) than the original study (45%) [7]. Accordingly, the average number of daily steps found in the present study (5.850) was slightly higher than in the two other quadrant studies (4.421 [12] vs. 5.521 [7]) and in most other COPD studies with an objective PA assessment (4.579) [47]. Notably, we identified these values and quadrant distributions despite the fact that the research took place in the setting of inpatient rehabilitation and that the individuals of the present sample exhibited more severely impaired lung function (FEV_1_%pred: 40) than the individuals in the other quadrant studies coming from usual outpatient care (FEV_1_%pred: 44 [12] vs. 56 [7]). However, a considerable portion of participants were still involved in working life (76.6%); hence, the sample was significantly younger (mean age: 58 years) than those in other studies using the concept (mean age: 63 years [12] and 63 years [7]). These sample characteristics may have caused the trend toward more favorable levels of PC.

The present study has some limitations. First, we chose an exploratory approach for the identification of psychological variables. Accordingly, we did not apply corrections for multiple testing and did not specify any a-priori hypotheses for the analyses. Due to the exploratory nature of the investigation, it would be worthwhile to reconfirm the relevance of the most important factors identified in this analysis. Second, in line with the first limitation, the selection and examination of psychological variables could be rooted more strongly in theoretical considerations, for example, in established behavior change concepts from health psychology or sport and exercise psychology. Third, there was a short temporal delay of approximately 1 week between the end of the initial accelerometer measurement (T0) and the 6-min walking test at the beginning of the clinic stay (T1). Fourth, all data stemmed from one specific inpatient PR clinic in Germany. This affects the generalizability of the present findings. Fifth, the number of cases in the “can’t do” groups was relatively low, which may have underestimated the role of some psychological factors in the comparisons between the two subgroups. Sixth, validations of the COPD-specific FAB questionnaire have only been reported in a conference contribution [35] but not in a peer-reviewed main article. Lastly, this study is cross-sectional in nature. Longitudinal analyses would have added value to the present topic, for instance, by examining changes in quadrant affiliation over time or by separately investigating the long-term outcomes of each quadrant. Due to the different focus of the analyses and the extensive material, however, such analyses should be subject of separate reporting.

In addition to the contributions that the STAR study could make to the recent discussions in the field, we also intend to highlight some conceptual and methodological considerations regarding the “can do, do do” concept. In general, we appreciate that researchers recognized the important, yet not exhaustive role of PC in predicting PA levels, and have welcomed discussions on behavioral mechanisms [13, 14]. However, it must be noted that psychological mechanisms are not only relevant when examining PC–PA discrepancies but also play a role when assessing PC. Specifically, it was discussed that the distance achieved in the six-minute walking test depends on motivational factors and pacing strategies [48, 49]. Furthermore, the concept dichotomizes individuals along two dimensions simultaneously, based on fixed cut-off values. Accordingly, in our dataset, the group formation decreased the variance of the continuous PA and PC variables by 45% and 49%, respectively. In this regard, other empirical classification procedures such as cluster analyses [8] or latent class analyses [50] might handle this problem better. From a conceptual perspective, the dichotomy also entails the risk that individuals are increasingly categorized into “the handicapped” (“can’t do”) on the one hand, and into “the healthy” (“can do”) on the other. Due to their divisive and thus stigmatizing potential, such clear-cut and binary assumptions are often challenged by health science [51]. Against this backdrop, an advancement of the existing approach may be helpful to support the broad acceptance of such a concept within the academic and professional fields of pulmonology. For instance, it could be worthwhile to employ person-centered analytical strategies while simultaneously considering disease-related, PA-related, psychological, and competence-oriented variables. Nevertheless, it appears that the PC–PA quadrant concept has the potential to benefit the daily clinical practice for individuals with COPD [6].

## Conclusions

To the best of our knowledge, this is the first study that extensively links psychological variables to the PC–PA quadrants conceptualized for individuals with COPD. The exploratory regression models provided preliminary evidence that disease-specific anxiety and fear avoidance behaviors may explain differences in PA behavior across all levels of PC. While depression emerged as an additional variable in individuals with lower relative PC, competence-oriented indicators additionally predicted PA levels in COPD rehabilitants with higher relative PC. Even though our results may serve as a point of departure for deriving subgroup-specific implications for exercise-based interventions, the present findings warrant confirmation through further studies.

## Abbreviations

COPD: Chronic Obstructive Pulmonary Disease
PC: Physical Capacity
PA: Physical Activity
STAR: Stay Active After Rehabilitation [study]
PAHCO: Physical Activity-related Health Competence
